# Non-invasive brain stimulation for neuropathic orofacial pain: a mini-review

**DOI:** 10.3389/fpain.2026.1746977

**Published:** 2026-04-15

**Authors:** Xiaoying Lin, Xiaomin Niu, Ting Hu, Jun Zhang, Yingxiu Diao, Jinqian Li, Qinjie Yang, Chunyang Liao, Tiantian Xin, Shuqin Li, Xiaying Fu, Ketao Du, Jianghua Cheng

**Affiliations:** 1 Department of Rehabilitation Medicine, South China Hospital, Medical School, Shenzhen University, Shenzhen, Guangdong, China; 2 General Administration of Customs (Beijing) International Travel Health Care Center, Beijing, China; 3 Gannan Medical University, Ganzhou, Jiangxi, China; 4 Department of Rehabilitation Medicine, Dongguan Huangjiang Hospital, Dongguan, Guangdong, China

**Keywords:** atypical facial pain, burning mouth syndrome, neuropathic orofacial pain, post-herpetic neuralgia, transcranial direct current stimulation, transcranial magnetic stimulation, trigeminal neuralgia

## Abstract

Neuropathic orofacial pain (NOP) can seriously affect the quality of life of patients. Due to the concentration of pain in the central area of the craniofacial region, it is not only highly invasive but also easily distracts the patient's attention, resulting in its destructive nature far exceeding that of distal limb pain. As emerging non-invasive therapies, Repetitive Transcranial Magnetic Stimulation (rTMS) and Transcranial Direct Current Stimulation (tDCS) are bringing hope for the treatment of NOP. This brief review summarizes existing evidence on their efficacy, highlighting that pain phenotype may be a key determinant of treatment response and warrants further investigation. High-frequency (10–20 Hz) rTMS over the primary motor cortex (M1) reduces trigeminal and postherpetic neuralgia pain by 30%–45%, with effects lasting weeks to months. Non-somatotopic hand M1 stimulation appears to produce comparable facial analgesia via descending pain pathways. For tDCS, preliminary evidence suggests pure paroxysmal pain may respond more robustly than persistent pain, implicating central sensitization as a potential negative predictor. Current evidence is limited by small samples and heterogeneous protocols. Future research should prioritize phenotype-stratified trials, optimal parameters, and synergy with pharmacotherapy.

## Background

**Figure 1:**
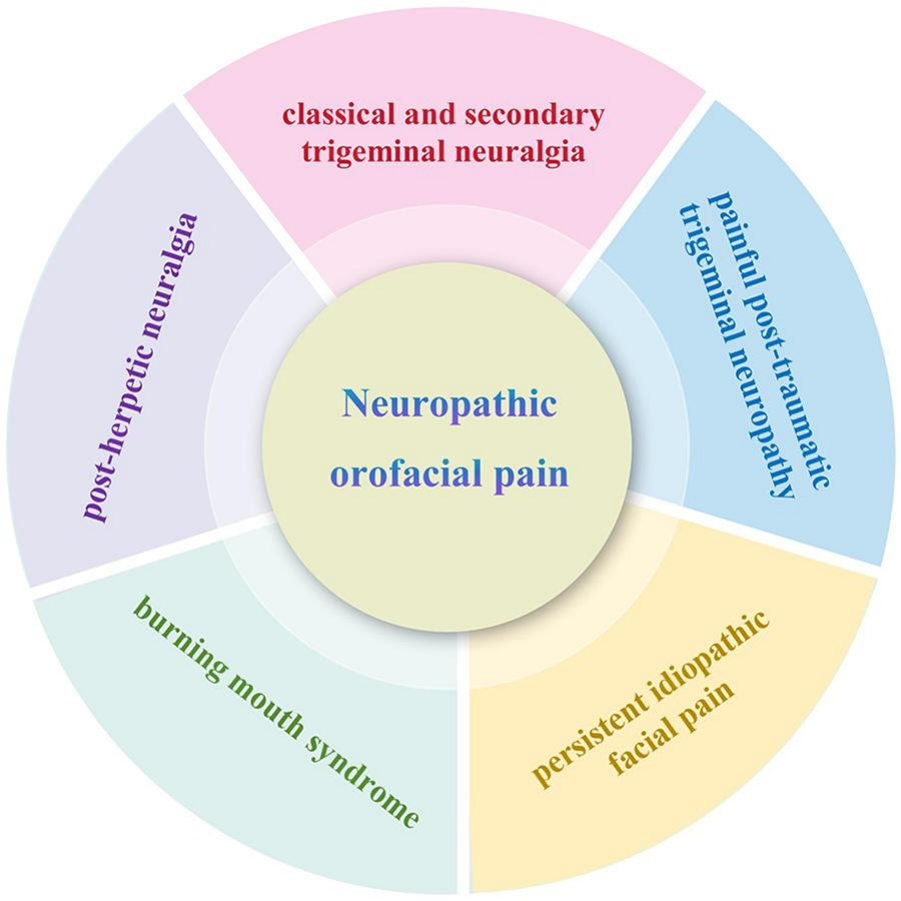
Clinical phenotypes of NOP.

Neuropathic Orofacial Pain (NOP) is a distinctive pain syndrome deriving from a lesion or dysfunction of the somatosensory pathways supplying the orofacial region (1). Studies have demonstrated that 19%–26% of adults suffer from orofacial pain within a month, with some studies reporting rates as high as 48%, while the prevalence of chronic oro-facial pain is approximately 8%–15% (2). NOP includes a series of related diseases, authoritatively classified under “painful cranial neuropathies and other facial pains” in the International Classification of Headache Disorders, 3rd edition (ICHD-3) (3). Key clinical phenotypes within this classification consist of classical and secondary trigeminal neuralgia (TN), painful post-traumatic trigeminal neuropathy, and post-herpetic neuralgia. Besides, disorders such as burning mouth syndrome and persistent idiopathic facial pain, once considered idiopathic, are now classified as distinct NOP phenotypes, with clinical neurophysiological evidence confirming underlying somatosensory nervous system dysfunction (2). Figure 1 shows the clinical phenotypes of NOP.

Considerable variation in individual presentations and treatment responses persists among these phenotypes. The pathophysiological underpinnings of NOP are complex and not yet fully elucidated (4). Peripheral nerve injury can trigger spontaneous ectopic discharges, which are interpreted by the brain as real pain (5, 6). Persistent afferent barrage may induce central sensitization—characterized by amplified nociceptive signaling, expanded receptive fields, and enhanced pro-inflammatory cytokine release—leading to sustained neuroinflammation and impaired descending inhibition (7–9). These mechanisms not only drive pain chronicity but may also underlie differential treatment responsiveness across patients. Routine analgesics (e.g., NSAIDs, opioids) are often ineffective, and first-line neuropathic pain agents (e.g., gabapentinoids, tricyclic antidepressants) are frequently limited by dose-related side effects, resulting in poor tolerability and low adherence (10). This has prompted interest in alternative or supportive therapies. Repetitive transcranial magnetic stimulation (rTMS) and transcranial direct current stimulation (tDCS) are non-invasive brain stimulation techniques that modulate cortical excitability and central network function. Emerging evidence suggests their potential efficacy in NOP, yet whether treatment response differs across pain phenotypes—such as paroxysmal vs. persistent pain—remains poorly understood and warrants further investigation. This mini-review critically synthesizes current evidence for rTMS and tDCS in managing NOP, with particular emphasis on pain phenotype as a potential predictor of treatment response and the neurophysiological mechanisms that may underlie such differences.

## Methods

We searched PubMed, Cochrane and Embase from the earliest records to 30 October 2025, combining terms for non-invasive brain stimulation (rTMS, tDCS) with keywords for NOP such as trigeminal neuralgia, burning mouth syndrome, atypical facial pain and post-herpetic neuralgia. Randomised controlled trials, open-label studies and case series published in English were included; animal studies, conference abstracts and reviews were excluded. Two authors independently screened the records; disagreements were resolved by a third reviewer. A narrative synthesis was used to summarise the findings.

### Pathophysiology of NOP

The pathophysiological mechanisms of NOP involve multiple interactions between the peripheral and central nervous systems and are significantly modulated by psychosocial factors, collectively forming a complex network model (5, 10).

The peripheral mechanism is the initial stage of pain, and peripheral nerve damage can cause changes in molecular mechanisms, leading to spontaneous ectopic firing of damaged neurons (5, 6). During this process, spontaneous and abnormal ectopic discharges are received and interpreted by the brain as real pain, leading to spontaneous pain in clinical practice, which becomes the root cause of neuropathic pain. The continuous influx of abnormal signals can cause excessive excitation of spinal cord and brainstem neurons, increased synaptic efficiency, and expanded receptive fields, known as central sensitization (6). The process of central sensitization amplifies the release of pro-inflammatory cytokines by activating microglia and astrocytes, forming a positive feedback loop (7), which increases the signal intensity of central sensitization and leads to persistent neuroinflammation due to impaired autophagy function (8), as well as weakened downward analgesic inhibition pathways originating from the brainstem, exacerbating pain perception (9). That is to say, once central sensitization occurs, normal and harmless tactile signals (such as light touch and wind blowing) transmitted through low threshold A β fibers will also be misinterpreted by the sensitized central system as severe pain (i.e., pain hypersensitivity). Meanwhile, the pain response to harmful stimuli can also be excessively amplified (i.e., hyperalgesia). On the basis of these neurobiological processes, psychosocial factors also play a role. Specifically, cognitive emotional factors such as anxiety, depression, and pain catastrophizing can affect the function of the prefrontal limbic system, amplify pain signals, increase treatment resistance, and ultimately consolidate clinical symptoms (11). Importantly, these pathophysiological processes—particularly central sensitization—may manifest clinically as distinct pain phenotypes (e.g., paroxysmal vs. persistent pain), which could in turn influence responsiveness to neuromodulation.

### Application of rTMS in NOP

Currently, high-frequency (10 Hz) rTMS targeting the primary motor cortex (M1) is the most consistent intervention for NOP.

Beyond the standard 10 Hz protocol, studies have also examined higher frequencies to optimize analgesic efficacy. Fricová et al. conducted a randomized trial comparing 10 Hz and 20 Hz rTMS in 59 patients with chronic orofacial pain, demonstrating that 20 Hz stimulation (720 pulses/session, 95% motor threshold, 5 sessions) produced superior pain reduction compared to 10 Hz (600 pulses/session), with significant improvements in both VAS scores and tactile thresholds (von Frey filaments) (12). Similarly, Pei et al. directly compared 5 Hz and 10 Hz rTMS in 60 patients with postherpetic neuralgia, finding that 10 Hz was significantly more effective than 5 Hz (39.89% vs. 28.38% VAS reduction, *P* = 0.022), with benefits persisting at 3-month follow-up (13). These findings suggest a frequency-dependent efficacy gradient, with 10–20 Hz range offering optimal analgesia, though higher frequencies may carry trade-offs in tolerability.

Multiple randomized controlled trials have demonstrated that 10 Hz rTMS applied to the M1 contralateral to the pain side can significantly reduce pain intensity in NOP (with average Visual Analogue Scale reductions of 30%–45%), achieving response rates of 50%–65%, and sustaining effects for up to 3 months post-treatment (14, 15). This pain-relieving effect was also confirmed by a recent randomized sham-controlled study on intractable postherpetic neuralgia (16). Notably, significant facial pain relief can be achieved even when stimulating the hand area of M1. Consistent with this observation, Functional Magnetic Resonance Imaging (fMRI) studies have demonstrated that motor cortex stimulation modulates activity in remote pain-modulatory subcortical structures, including the thalamus and periaqueductal gray (15, 17).

Evidence regarding the necessity of somatotopic targeting in M1-rTMS for facial pain remains inconsistent. Ayache et al. (18) conducted a crossover trial in 66 patients with neuropathic pain and found that a single session of high-frequency rTMS—whether navigated to the cortical representation of the painful area or non-navigated to the hand motor hotspot—significantly reduced upper and lower limb pain, but did not reach statistical significance for facial or hemibody pain (19). The authors attributed this negative finding to the small sample size of facial pain patients (*n* = 16) and possible inclusion of non-responders, noting that prior studies had demonstrated analgesic effects of rTMS on facial pain (19). In contrast, André-Obadia et al. (19) directly compared hand versus face motor area stimulation in 32 patients with neuropathic pain of the face or upper limb. They found that stimulation of the hand motor area—but not the face area—produced significant pain reduction for both upper limb and facial pain, leading the authors to conclude that strict somatotopic targeting is not warranted and that the hand motor area, being technically easier to localize, may be the preferred target regardless of pain location (20). These conflicting findings highlight the need for larger, phenotype-stratified trials to clarify the role of somatotopic targeting in orofacial pain. The concept of “non-somatotopic stimulation”—wherein hand motor area stimulation engages descending pain inhibitory pathways that project to both limb and facial nociceptive circuits—has been proposed to explain analgesic effects observed with hand M1 rTMS in limb pain (19) and with hand M1 tDCS in trigeminal neuralgia (21).

Integrating neuroimaging findings with prior animal experimental evidence, Khedr et al. further proposed a complete pathway model: M1 excitation enhances inhibition on the spinal trigeminal nucleus via corticoreticular synapses (17). Notably, significant facial pain relief can be achieved even when stimulating the hand area of M1. This so-called “non-somatotopic” analgesic effect is thought to arise not from somatotopic correspondence, but from activation of descending pain modulatory pathways. Specifically, stimulation of the hand motor area is believed to engage corticothalamic-brainstem projections that inhibit nociceptive transmission from both limb and facial territories (17). This convergent evidence suggests that non-somatotopic stimulation may achieve facial analgesia via these descending inhibitory pathways, rather than relying on precise anatomical correspondence.

Moreover, pain phenotype appears to distinctly influence treatment response. Although dedicated phenotype-stratified analyses in rTMS trials remain scarce, indirect observations suggest that patients with paroxysmal facial pain may derive greater benefit than those with persistent background pain (18, 19). This pattern is consistent with direct evidence from tDCS (20), lending preliminary support to the hypothesis that pain phenotype may modulate treatment response across NIBS modalities—a question warranting prospective investigation.

Novel stimulation protocols have been explored to enhance efficacy and reduce treatment duration. Theta burst stimulation (TBS), a patterned rTMS protocol delivering bursts of 3 pulses at 50 Hz repeated at 5 Hz, can induce long-term potentiation-like effects with shorter application times. However, Kohútová et al. randomized 19 patients with chronic orofacial pain to receive either intermittent TBS (iTBS) or sham TBS over M1, finding that while iTBS produced significant immediate pain reduction (VAS decrease 2.3 points, *P* = 0.02), this effect was transient and did not persist at 2-week follow-up (22). Quantitative sensory testing showed no significant changes in thermal or tactile thresholds. The authors concluded that single-session TBS is insufficient for lasting analgesia, reinforcing the importance of the “dose-time integral”—the combination of total pulses and treatment duration—for inducing durable neuroplastic changes (21, 23). Existing evidence suggests that multiple treatment sessions—rather than a single session—are required to induce durable neural plasticity changes and achieve sustained analgesic effects (21, 23).Studies indicate that a protocol delivering 3000 pulses daily over 10 days, with each session lasting at least 20 min, is optimal for treating post-herpetic neuralgia and trigeminal neuropathies (21, 23).

Beyond M1, some studies have explored the secondary somatosensory cortex (S2) and dorsolateral prefrontal cortex (DLPFC) as potential targets. For S2 stimulation, patients' sensory-discriminative pain dimension scores decreased significantly, along with reduced functional connectivity between the anterior insula and putamen (24–26). Combining fMRI connectivity analysis, the authors speculated that S2 excitation achieves analgesia by inhibiting the hyper-synchronization of the insular-basal ganglia circuit, thereby reducing the amplification of orofacial pain discriminative signals. As for the DLPFC stimulation, high-dose stimulation led to enhanced top-down inhibition in the mesothalamic-cingulate pathway, restored imbalance in the anterior cingulate-insula functional connectivity, and the degree of analgesia was unrelated to mood scores (27–29). This indicates that the DLPFC exerts its analgesic effect primarily by remodeling the central pain matrix rather than through an antidepressant mechanism. The characteristics of included rTMS and TBS studies are summarized in Tables 1 and 2.

**Table 1:** Characteristics of rTMS and TBS studies in neuropathic orofacial pain.

Study	N	Pain (Type-Phenotype)	Target	Parameters	Key Results	F/U
Lefaucheur 2004 (33), France	60 (30/30)	Neurogenic-face/UL/LL	M1-hand	10 Hz, 80%RMT, 1000p, 1s	VAS↓22.9% vs. sham; M1-hand↓facial pain	<5min
Khedr 2005 (17), Egypt	28	Periph/Cent NP-Parox	Motor cortex	Daily sessions (NS)	Long-lasting effect	NS
Fricová 2013 (12), Czech	59 (36/23)	Chronic OFP-Mixed	M1	10/20 Hz, 95%MT, 600-720p/s, 5d	20Hz > 10 Hz (*p* = 0.038)	2wk
Ma 2015 (14), China	40 (20/20)	PHN-Persist	M1	10 Hz, 3000p/d, 10d	VAS↓sustained 3mo	3mo
Lindholm 2015 (26), Finland	8	Drug-resist NOP-Mixed	R-S2	10 Hz, 2000p/d, 5d	6/8 parox-dominant→better	1mo
Umezaki 2016 (29), USA	20 (12/8)	BMS-Persist	DLPFC	10 Hz, 110%RMT, 3000p, 10d	VAS↓67% at 60d	2mo
Ayache 2016 (18), France	66	NP-face/hemi/UL/LL-Persist	M1/non-nav	10 Hz, 90%RMT, 3000p, 1s	Limb pain↓; facial pain NS	1wk
Kohútová 2017 (22), Czech	19 (10/9)	Chronic OFP-Persist	M1	iTBS, 90%MT, 600p, 1s	VAS↓post (*p* = 0.02), lost at 2wk	2wk
André-Obadia 2018 (19), France	32 (20/12)	NP-UL/face-Persist	Hand vs. Face-M1	20 Hz, 90%MT, 1600p/s	Hand-M1: facial + limb pain↓; Face-M1: NS	5d
Pei 2019 (13), China	60 (20/20/20)	PHN-Persist	M1	5/10 Hz, 80%MT, 1500p/d, 15d	10Hz:↓39.9% (*p* = 0.022), sustained 3mo	3mo
Wang 2023 (16), China	60 (20/20/20)	PHN-Persist	M1/DLPFC	10 Hz, 100%RMT, 3000p/d, 10d	M1: VAS↓sustained 3mo	3mo

BMS, burning mouth syndrome; DLPFC, dorsolateral prefrontal cortex; iTBS, intermittent theta burst stimulation; LL, lower limb; M1, primary motor cortex; MT, motor threshold; N, total sample size (active/sham or intervention/control); non-nav, non-navigated; NS, not specified; OFP, orofacial pain; Parox, paroxysmal; Persist, persistent; PHN, postherpetic neuralgia; p, pulses; RMT, resting motor threshold; R-S2, right secondary somatosensory cortex; s, session; TN, trigeminal neuralgia; UL, upper limb; VAS, visual analogue scale.

**Table 2:** Characteristics of tDCS studies in neuropathic orofacial pain.

Study	N	Pain (Type-Phenotype)	Target	Parameters	Key Results	F/U
Antal 2010 (30), Germany	16	Chronic TN-Persist	L-M1	1 mA, 20 min, 5d	VAS↓37%, 63% resp; persistent pain responded	28d
Hagenacker 2014 (20), Germany	10	Classical TN-Parox	M1-hand	1 mA, 20 min, 14d	Pure parox:↓24.2% (*p* = 0.032); Mixed: 0%	1wk

L-M1, left primary motor cortex; M1-hand, hand area of primary motor cortex; M1, primary motor cortex; N, total sample size; Parox, paroxysmal; Persist, persistent; TN, trigeminal neuralgia; VAS, visual analogue scale.

### Application of tDCS in NOP

Compared to rTMS, research on tDCS in NOP is less abundant, but preliminary evidence is concentrated and positive.

The most representative study to date is a randomized double-blind crossover trial conducted by Hagenacker et al. (20). It showed that self-administered anodal tDCS (1 mA, 20 min/day, for 14 consecutive days) targeting the contralateral M1 significantly relieved paroxysmal pain in patients with classical TN (29% reduction in VRS score, *p* = 0.008), with one patient achieving complete pain freedom, while attack frequency showed no significant change. Critically, Hagenacker et al. performed a pre-specified subgroup analysis based on pain phenotype that revealed a striking dissociation: patients with purely paroxysmal pain (*n* = 6) showed significant VRS reduction (24.2%, *P* = 0.032), whereas those with concomitant persistent background pain (*n* = 4) showed absolutely no response (0% change, *P* = 1.0) (20). This dichotomy was mirrored in electrophysiological findings: only the paroxysmal pain group exhibited decreased pain-related evoked potential (PREP) amplitudes after tDCS (r = 0.71, *P* = 0.032), suggesting that central sensitization—marked by persistent pain and amplified nociceptive processing—renders patients refractory to tDCS. These findings suggest that pain phenotype, rather than diagnosis *per se*, may be a critical determinant of tDCS efficacy in trigeminal neuralgia.

Mechanistically, tDCS resulted in prolonged N2 latency and reduced peak-to-peak amplitude of the trigeminal PREP, suggesting inhibited central trigeminal nociceptive processing, although the nociceptive Blink Reflex (nBR) showed no significant change (20). The authors speculated that tDCS might indirectly inhibit central sensitization of facial pain by anodally enhancing M1 cortical excitability, modulating thalamocortical networks, and promoting endogenous opioid release. Notably, the anodal tDCS target was the hand M1 area, yet it still significantly relieved facial pain. Citing fMRI and anatomical tracing studies, the authors noted that hand and face M1 representations have functional connectivity and share common projections to the thalamic Ventroposteromedial nucleus (VPM) and midbrain Periaqueductal gray (PAG) (17, 20).

Additionally, Antal et al. in an early exploratory study found that consecutive 5-day anodal tDCS (1 mA, 20 min/day) targeting the left M1 significantly relieved chronic pain including TN, with a maximum pain reduction of 37%, effects lasting until day 28 post-stimulation, and 63% of patients defined as “responders” (≥30% VAS reduction) (30). Although this study did not perform a separate subgroup analysis for TN patients, the included TN patients all presented with persistent facial pain and responded well to tDCS. This suggests that tDCS might be effective for patients with persistent facial pain, such as those with TN. However, additional study is warranted to confirm this discovery.It should be noted, however, that Antal et al. did not explicitly distinguish between continuous background pain and frequent paroxysmal attacks; their use of “persistent pain” may therefore differ from the definition used in Hagenacker et al. (20).

This apparent contradiction between Antal et al.'s findings and Hagenacker et al.'s observation that persistent pain predicts poor tDCS response may reflect several methodological differences. First, Antal et al. employed a shorter stimulation course (5 days vs. 14 days in Hagenacker et al.), raising the possibility that treatment duration interacts with pain phenotype to determine efficacy. Second, Hagenacker et al. specifically examined classical trigeminal neuralgia with concomitant persistent background pain—a phenotype indicative of advanced central sensitization—whereas Antal et al. included TN patients without detailed phenotypic characterization, potentially encompassing earlier disease stages with less established central plasticity. Third, the “persistent pain” in Antal's cohort may represent ongoing paroxysmal attacks rather than continuous background pain, highlighting the importance of precise phenotype definitions. These discrepancies underscore the need for standardized pain phenotype classification in future tDCS trials.

In summary, tDCS performs well in the treatment of neuropathic pain, demonstrating mild to moderate analgesic effects, with pain phenotypes, stimulus parameters, and target selection playing important roles in the efficacy. Compared with rTMS, tDCS has multiple advantages such as portability, ease of operation, and economic feasibility. Therefore, it is worth considering whether it can play the role of a synergistic therapy or alternative during rTMS treatment. However, tDCS currently lacks evidence from large-scale randomized controlled trials, with poor parameter consistency and unclear efficacy prediction indicators. In the future, personalized parameter optimization schemes and mechanisms of action need to be explored.

### Methodological quality and certainty of evidence

The evidence summarized in this review is subject to important methodological limitations. Most included RCTs had small sample sizes (median N per active arm < 25) and were underpowered to detect subgroup effects or rare adverse events. Sham-controlled designs were used in the majority of studies, but adequacy of blinding—particularly for rTMS—was seldom formally assessed. Follow-up durations were highly variable, ranging from minutes to three months, and only a few studies reported outcomes beyond the immediate post-stimulation period. Heterogeneity in stimulation parameters (frequency, pulse number, session count, coil type, navigation use) precludes meta-analytic pooling and limits direct comparability across studies. According to the GRADE framework, the overall certainty of evidence for both rTMS and tDCS in NOP is low to very low, due to risk of bias, imprecision, and inconsistency (3, 21). Future trials should adhere to recommended reporting standards (e.g., CONSORT for rTMS/tDCS) and incorporate prospective registration, blinded outcome assessment, and *a priori* stratification by pain phenotype.

## Discussion

The analgesic effects of rTMS and tDCS in NOP summarized above align with recent comprehensive reviews on non-invasive neuromodulation for headache and craniofacial pain conditions (31). These reviews have emphasized that responder profiling, stimulation durability, and phenotype-specific targeting are key determinants of clinical outcomes—a framework that resonates strongly with the findings of the present synthesis. However, while the headache literature has focused largely on migraine prophylaxis and central sensitization in the context of attack frequency, the NOP literature offers a unique opportunity to examine pain phenotype (paroxysmal vs. persistent) as a direct, clinically observable proxy for central sensitization. Moreover, the emerging emphasis on combination treatment paradigms in migraine prevention (32) raises the question of whether rTMS or tDCS could be similarly integrated with pharmacotherapy in NOP—an area that remains virtually unexplored (31, 32).

Multiple studies have demonstrated that high-frequency rTMS targeting the M1 area is one of the most effective non-invasive brain stimulation (NIBS) techniques for neuropathic pain (21, 30, 33). At the same time, tDCS has shown great potential in the field of home treatment due to its unique advantage of combining convenience with cost-effectiveness. The efficacy of both rTMS and tDCS in NOP is modulated by stimulation parameters such as frequency, pulse number, and treatment duration (21). Importantly, pain phenotype—whether paroxysmal or persistent—has emerged as an independent predictor of treatment response, with direct evidence from tDCS trials and converging observational support from rTMS studies (18–20). These findings align with the broader shift toward personalized neuromodulation strategies in pain medicine, and parallel recent efforts to develop combination treatment paradigms in headache prophylaxis (32).

However, the current evidence base is constrained by several methodological limitations: sample sizes remain small, sham-controlled designs are underutilized, follow-up durations are often insufficient, and stimulation protocols are highly heterogeneous across studies. These issues warrant attention in future research.

Future research on NIBS in NOP should prioritize several interconnected directions. First, phenotype-driven trial designs are urgently needed: future RCTs should stratify randomization and analysis by pain phenotype (paroxysmal vs. persistent) *a priori*, rather than pooling heterogeneous populations. Enrichment strategies that selectively enroll patients with pure paroxysmal pain may increase effect sizes and reduce sample size requirements. Second, direct head-to-head comparisons are required to establish optimal stimulation protocols, including target (hand M1, face M1, S2, DLPFC), frequency (10 Hz, 20 Hz, iTBS), session number, and pulse dose per session; protocol standardization would facilitate meta-analytic synthesis and clinical translation. Third, predictive biomarkers should be prospectively evaluated, encompassing neurophysiological measures such as PREP, nBR, and SICI, as well as neuroimaging markers like resting-state fMRI connectivity. Biomarkers of central sensitization—for instance, temporal summation or conditioned pain modulation—may help identify patients most likely to benefit from or fail NIBS. Finally, the potential synergy between NIBS and pharmacotherapy warrants exploration: preclinical and early-phase clinical studies should examine whether rTMS or tDCS can enhance the effects of first-line neuropathic pain agents (e.g., gabapentinoids, tricyclic antidepressants), reduce required drug dosages, or mitigate side effects. Such combination strategies, already showing promise in migraine, remain virtually unexplored in NOP and represent a priority for future investigation.

## Conclusion

As promising treatment options in the field of neuropathic pain management, rTMS and tDCS bring bright prospects for the management of NOP. Existing evidence shows that their safety is good and their efficacy is emerging, especially in patients with paroxysmal pain who have achieved remarkable success. However, further optimization of treatment protocols and validation of long-term efficacy in diverse patient populations are still required, underscoring the need for more high-quality research.
